# Selector-free resistive switching memory cell based on BiFeO_3_ nano-island showing high resistance ratio and nonlinearity factor

**DOI:** 10.1038/srep23299

**Published:** 2016-03-22

**Authors:** Ji Hoon Jeon, Ho-Young Joo, Young-Min Kim, Duk Hyun Lee, Jin-Soo Kim, Yeon Soo Kim, Taekjib Choi, Bae Ho Park

**Affiliations:** 1 Division of Quantum Phases & Devices, Department of Physics, Konkuk University, Seoul 143-701, Korea; 2 HMC, Department of Nanotechnology and Advanced Materials Engineering, Sejong University, Seoul 143-747, Republic of Korea; 3 IBS Center for Integrated Nanostructure Physics (CINAP), Institute for Basic Science, Sungkyunkwan University, Suwon 440-746, Republic of Korea; 4 Department of Energy Science, Sungkyunkwan University, Suwon 440-746, Republic of Korea

## Abstract

Highly nonlinear bistable current-voltage (*I–V*) characteristics are necessary in order to realize high density resistive random access memory (ReRAM) devices that are compatible with cross-point stack structures. Up to now, such *I–V* characteristics have been achieved by introducing complex device structures consisting of selection elements (selectors) and memory elements which are connected in series. In this study, we report bipolar resistive switching (RS) behaviours of nano-crystalline BiFeO_3_ (BFO) nano-islands grown on Nb-doped SrTiO_3_ substrates, with large ON/OFF ratio of 4,420. In addition, the BFO nano-islands exhibit asymmetric *I–V* characteristics with high nonlinearity factor of 1,100 in a low resistance state. Such selector-free RS behaviours are enabled by the mosaic structures and pinned downward ferroelectric polarization in the BFO nano-islands. The high resistance ratio and nonlinearity factor suggest that our BFO nano-islands can be extended to an N × N array of N = 3,740 corresponding to ~10^7^ bits. Therefore, our BFO nano-island showing both high resistance ratio and nonlinearity factor offers a simple and promising building block of high density ReRAM.

With the recent revolution in mobile device technology and the increasing need for higher data storage density, non-volatile memory technologies have rapidly developed^1^,^2^,^3^,^4^,^5^. Among various non-volatile memories, resistive random access memory (ReRAM) based on metal oxide has emerged as a promising alternative to flash memory due to its high speed, long retention time, good endurance, low operating voltage, and high scalability^1^,^2^,^3^,^4^,^5^. However, achieving high memory performance and overcoming the scaling limitations remain significant challenges and require nanoscale device structures, reliable resistive switching (RS) materials, and cost-effective device architectures.

Recently, stackable cross-point arrays and three-dimensional (3D) vertical device configurations have been proposed as advanced device architectures for achieving ultra-high density^6^. Compared to conventional transistor-accessed memory cells, two-terminal nanostructured RS devices that form cross-points without using access devices are scalable down to 4F^2^ for the smallest cell size, where F is the minimum feature size^7^. Moreover, such cross-point arrays could be fabricated in multilayers for 3D memory to further increase storage density. However, unwanted leakage current paths through neighbouring cells (a well-known sneak path problem in cross-point structures) must be addressed because such paths can give rise to misreading of memory states^8^. To eliminate sneak current, various selection elements, e.g. diodes, threshold devices, and metal-insulator switching devices, can be connected to the memory elements in series^7^,^9^,^10^,^11^,^12^,^13^,^14^,^15^ leading to more complex device structures and fabrication processes. These devices are based on highly nonlinear current-voltage (*I*–*V*) characteristics whose conduction current levels strongly depend on the applied voltage, and are typically made of metal oxides (often with nonstoichiometric oxygen) including titanium-oxide^12^, tantalum-oxide^9^,^15^, vanadium oxide^16^, *p*-CuO/*n*-InZnO heterojunctions^10^, and nickel-oxide^17^. Realizing selector-free memory cell in a simple metal-oxide-metal structure at the nanoscale is critical for the future implementation of high-density cross-point ReRAM that is free from the sneak path problem. Selector-free memory cell significantly reduces the leakage current in the idle state and thus cell-to-cell disturbance enabling larger array size and lower process complexity, in which both highly nonlinear and bistable *I–V* characteristics are required^15^,^16^,^17^,^18^,^19^.

In this respect, we need to design a nanoscale metal-oxide-metal structure which contains two independent factors causing high nonlinearity and non-volatile bistability of the *I–V* characteristics, respectively. Because ferroelectric polarization has inverse bound charges at its tail and head, a metal-ferroelectric-metal structure usually has asymmetric top and bottom interfaces and thus shows highly nonlinear rectifying *I–V* curves^20^. In contrast, ionic defects, such as oxygen vacancies, can give rise to bistable *I–V* curves through their migration induced by the external bias^21^. Therefore, a defective ferroelectric capacitor incorporating ferroelectric polarization and ionic defects is a good candidate for the selector-free memory cell.

In general, most ferroelectric materials with wide bandgaps have been seldom considered as selector elements because of their poor charge conduction^22^. In contrast, BiFeO_3_ (BFO) is a semiconducting ferroelectric material with a narrow bandgap (~2.7 eV) as well as large polarization (~60 μC/cm^2^)^23^,^24^. The narrow bandgap and large polarization of semiconducting BFO can give rise to high forward current and nonlinearity factor in a selector-free memory cell.

Here, we report selector-free RS behaviours in BFO nano-island arrays probed by using conductive atomic force microscopy (C-AFM). We observe mosaic nano-crystalline structures in a BFO nano-island grown on a 0.5 wt% Nb-doped SrTiO_3_ (Nb:STO) substrate, which has pinned downward polarization. *I–V* curves obtained from each BFO nano-island show both bipolar RS with resistance ratio of 4,420 and highly nonlinear behaviour with nonlinearity factor α of 1,100 at a reading voltage (V_read_) of 3.1 V (the ratio of resistance at 1/2 V_read_ to the resistance at V_read_ in a low resistance state). Under the condition of 10% normalized read margin, such high resistance ratio and nonlinearity factor suggest that our BFO nano-islands can be extended to an array (N rows × N columns) of N = 3,740. Because this large array size corresponds to ~10^7^ bits, the selector-free memory cell based on BFO nano-island can become a simple building block of high density ReRAM free from the sneak path problem.

## Results and Discussion

**Figure 1:**
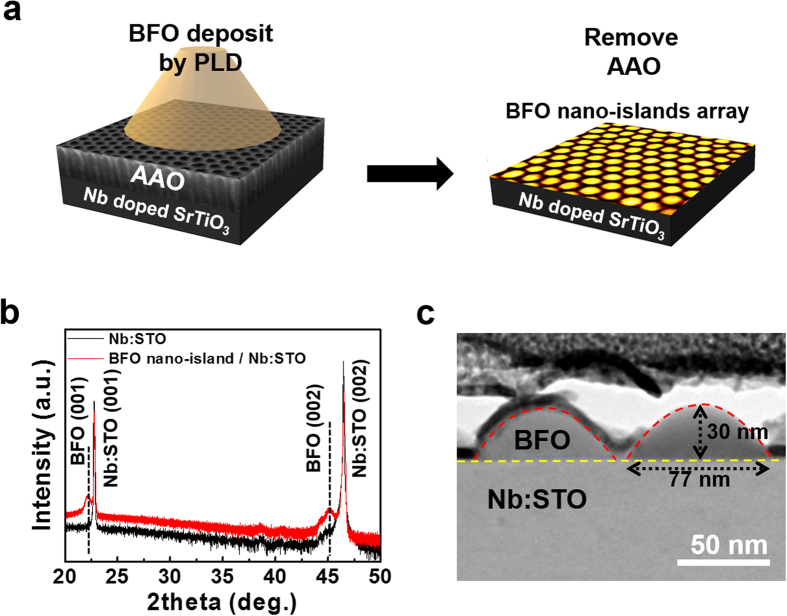
High density array of BiFeO_3_ nano-islands. (**a**) The fabrication procedure of BFO nano-islands array illustrated by a SEM image of AAO and AFM topography image of BFO nano-islands. (**b**) High-resolution XRD patterns. (**c**) TEM cross-section image of BFO nano-islands on Nb:STO (100) substrate. The average diameter of the dome-shaped nano-islands is almost identical to the pore size of the AAO template.

Figure 1a illustrates the fabrication process of high density array of BFO nano-islands on a Nb:STO (100) single crystal substrate through an anodized aluminum oxide (AAO) template by using pulsed laser deposition (PLD). The structures of the AAO template and BFO nano-island array are illustrated using scanning electron microscopy (SEM) and AFM topography images. The structural characterization of the BFO nano-islands was performed using high-resolution X-ray diffraction (XRD) and cross-sectional transmission electron microscopy (TEM). High-resolution XRD data (Fig. 1b) show that the BFO nano-islands have highly (001)-oriented structures with an out-of-plane lattice constant of ~0.400 nm, which is slightly larger than the bulk pseudo-cubic BFO lattice constant (0.396 nm). This increase in the out-of-plane lattice constant arises either from compressive stress imposed through a large lattice mismatch with the Nb:STO substrate (0.390 nm) or from volume expansion of the lattice induced by the formation of oxygen vacancies^25^. Generally, the introduction of oxygen vacancies into BFO grown by PLD is associated with low oxygen partial pressure and Bi-rich target material used during deposition. From the cross-sectional TEM image of BFO nano-islands (Fig. 1c), the average diameter and height of the nano-islands are estimated to be ~77 nm and ~30 nm, respectively.

**Figure 2:**
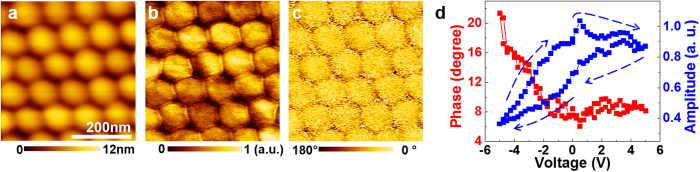
PFM study of BFO nano-islands. (**a**) AFM topography, (**b**) PFM amplitude, and (**c**) PFM phase images of BFO nano-islands. (**d**) Dependence of the PFM phase (red) and PFM amplitude (blue) on the applied voltage as swept between −5 V and +5 V, which are measured at a point on a single BFO nano-island. Polarization switching does not occur even with a high applied voltage.

The surface morphology and ferroelectric domain structure of the BFO nano-island array were probed using AFM topography and piezoresponse force microscopy (PFM), respectively. AFM topography image (Fig. 2a) reveals the presence of high-density and closely-packed array of BFO nano-islands. PFM amplitude image (Fig. 2b) exhibits almost a single contrast over the array of nano-islands, implying that the individual BFO nano-islands have single domain structures with similar polarization values. Domain boundaries appearing with considerably reduced PFM amplitudes are not observed in each BFO nano-island. PFM phase image (Fig. 2c) also confirms a single domain structure with a downward polarization (bright contrast) in each BFO nano-island. The piezoresponse hysteresis loop (Fig. 2d) measured at a point on a single BFO nano-island demonstrates its pinned local domain switching behaviour. Ferroelectric polarization switching generally appears as both a flipping by 180° in the phase of piezoresponse and a butterfly feature in the amplitude of piezoresponse, which are accompanied by hysteretic behaviour. For our BFO nano-island, however, the piezoresponse phase signal changes by only 12° when the external bias applied to a PFM tip varies from 5 V to −5 V and returns to its initial value shortly after turn-off of the external bias. The piezoresponse amplitude signal of our BFO nano-island monotonically decreases when sweeping the external voltage from +5 V to −5 V. These piezoresponse data imply that a high applied bias of −5 V cannot induce polarization reversal from downward to upward in our BFO nano-island, but it can temporarily reduce the downward polarization amplitude. Note that the polarization switching cannot be observed even after applying high bias voltage (above −7 V), and the BFO nano-island was instead damaged by soft breakdown. Therefore, we can argue that polarization of the as-grown BFO nano-islands on Nb:STO is pinned downward.

For BFO thin films deposited on Nb:STO substrates, the Fermi level at the BFO/Nb:STO interface is below the surface charge neutrality level, resulting in a positively charged interface^24^. To be a stable system with neutral interface-trap charges, the Fermi level of BFO must approach the surface charge neutrality level at the BFO/Nb:STO interface, making the as-grown polarization state be downward. The positive polarization charges of the as-grown downward polarization state in BFO assist the reduction of unwanted positive trapped charges at the BFO/Nb:STO heterointerface^24^. Furthermore, strain gradient by misfit dislocation or defect distribution causes the flexoelectric effect^26^; the strain gradient and flexoelectric effect are dominant in thin epitaxial ferroelectric films which have non-switchable polarizations^27^. As the thickness of an epitaxial ferroelectric film increases above a critical thickness, the flexoelectric effect rapidly decreases because the strain gradient readily vanishes in a thick film^27^. The polarization in our BFO nano-island with ~30 nm thickness is pinned downward because of the flexoelectric effect below a critical thickness, which is supported by ferroelectric polarization reversal of a thicker BFO nano-island with ~50 nm thickness (Supplementary Information, Fig. S1).

**Figure 3:**
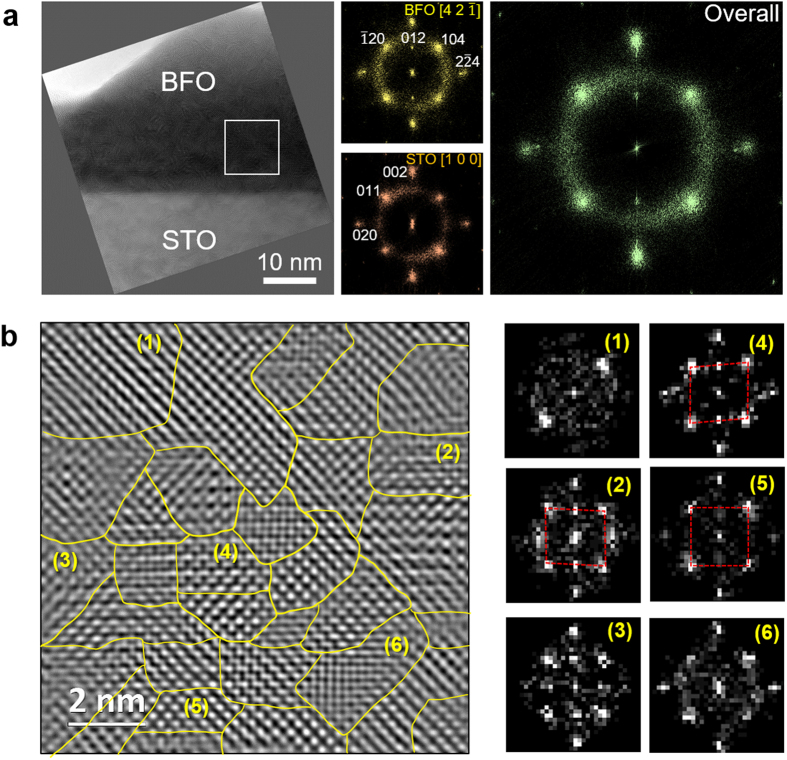
High-resolution TEM analysis of BFO nano-island grown on Nb:STO substrate. (**a**) Low-magnification image of the sample. Diffractograms that were obtained from fast Fourier transform (FFT) for the parts of BFO nano-island (yellow), Nb:STO substrate (dark pink), and the overall imaging region (green) are displayed on the right side panel. (**b**) High-resolution TEM image for the region marked with white box in (**a**). The individual BFO nano-grains are separated depending on their crystal orientations by yellow boundaries, and the corresponding FFT patterns to the individual nano-grains marked with numbers from 1 to 6 are represented on the right side panel.

Given that the BFO nano-island was identified as a highly-oriented structure based on the XRD data, the electrical properties obtained at the mesoscopic scale make it difficult to determine why this unique polarization pinning phenomenon occurs within the structure. Accordingly, we used high-resolution TEM (HR-TEM) to study the possible local deformations relevant to the microscopic origin of this behaviour in our highly-oriented BFO structures. A typical HR-TEM image of the BFO nano-islands is shown in Fig. 3a. We can see that the left part of a BFO hemisphere resides on the Nb:STO substrate. Fast Fourier transform (FFT) patterns of BFO (yellow) and Nb:STO (dark pink), equivalent to the electron diffraction patterns of these structures, show that the rhombohedral BFO (R3c) is epitaxially grown on the cubic Nb:STO (Pm 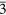 m) substrate with an orientation relationship of 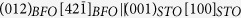. This interpretation is confirmed by the FFT pattern obtained for the whole image area (green) which reveals single crystalline BFO grown on Nb:STO in good agreement with the XRD analysis.

The BFO structure image obtained at this viewing direction contains pseudo-cubic structures oriented at [100] direction. It is worth noting that the in-plane and the out-of-plane axes at this orientation intersect at a right angle with a slight incline of ~0.5°. Atomic defects and low-angle grain boundaries in the BFO structure are expected when the epitaxial constraint between the BFO and the substrate becomes relaxed. The enlarged HR-TEM image of the BFO nano-island (Fig. 3b) reveals that the grown BFO sample is comprised of small nano-grains with slight mis-orientations, which are bordered by yellow lines. Note that the background noise in the HR-TEM image was removed by Fourier mask filtering to enhance the lattice image^28^. FFT patterns of selected nano-grains in Fig. 3b (displayed on the right panels) demonstrate that each grain is slightly tilted compared to the exact [100] BFO pseudo-cubic orientation, and furthermore the pseudo-cubic BFO lattices in the nano-grains are intrinsically sheared along both of the in-plane axes (see the FFT patterns of (2) and (4) in Fig. 3b). These small mismatches of crystal lattice and orientation between the nano-grains are not usually resolved on the mesoscopic scale, and could induce an increase in the strain energy of the system. Thus, we expect that the introduction of point and lattice defects such as oxygen vacancies, dislocations, and stacking faults in the grain boundaries of the system are necessary to accommodate the lattice strain. Using a geometric phase analysis (GPA) of the HR-TEM images to visualize how defect cores are placed inside the BFO nano-islands (Supplementary Information, Fig. S2), we clearly see that the defect cores are randomly distributed due to the mosaic configuration of the nano-grains within the sample. Taking the local structural characteristics of the BFO nano-islands into account, we find that each as-grown BFO nano-island is fully relaxed by the accommodation of multiple defect cores inside the structure, independent of any interface strain with the Nb:STO substrate. We can thus connect the oxygen vacancies in the system to a number of these defect cores, which may also be responsible for the exertion of pinning force on the domain wall motion and ferroelectric switching^29^. The small volume and low dimensionality of our BFO nano-island alleviates the energy cost of the downward polarization pinning. Local perturbations in BiFeO_3_ nano-islands, such as nano-grain boundaries and oxygen vacancies, can establish charge-carrier trapping centres and electrostatic potential barriers to electron migration like varistors^30^, which can eventually affect the charge conduction. Moreover, oxygen vacancy migration under electric field and oxygen vacancy coupling with ferroelectric polarization can provide intriguing resistive switching behaviour^31^,^32^.

**Figure 4:**
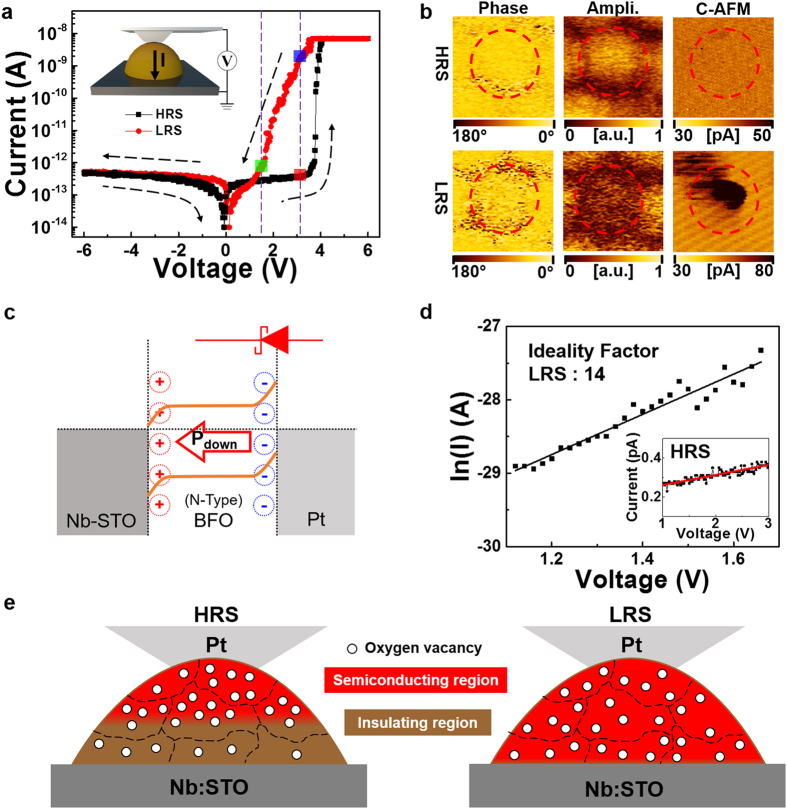
Highly nonlinear rectifying and bistable conduction behaviours. (**a**) *I*–*V* curves of Pt/BFO/Nb:STO at a voltage range between +6 V and −6 V. The inset shows the schematic illustration of measurement geometry. (**b**) PFM phase, PFM amplitude, and C-AFM images obtained from a single BFO nano-island at HRS and LRS states. (**c**) Schematic band diagram of a Pt/BFO/Nb:STO capacitor structure with downward polarization, which reveals the formation of a Schottky barrier at the top Pt/BFO interface. (**d**) ln(*I*) *vs V* data (solid dots) at LRS obtained from (**a**) and fitting results (line) with a Schottky conduction model. The inset shows the linear dependence of *I–V* data at HRS obtained from (**a**). (**e**) Schematic illustration of switching mechanism of our Pt/BFO/Nb:STO structure which can be considered as a serial combination of a Schottky diode formed at the top Pt/BFO interface and a bulk BFO resistor.

We measured local *I–V* characteristics of an individual BFO nano-island grown on an Nb:STO substrate at room temperature using a Pt/Ir coated AFM tip as a top electrode, as shown in Fig. 4a. Interestingly, we can observe both positive-forward rectifying behaviour with high turn-on voltage and bipolar RS. The initial transition from high resistance state (HRS) to low resistance state (LRS) occurs at high voltages (>+3.8 V). The resistance ratio of R_HRS_/R_LRS_ measured at V_read_ = 3.1 V (blue and red dots) reaches ~4,420. These observations were confirmed by measurements at different BFO nano-islands.

As shown in Supplementary Information Fig. S3a, we performed cyclic *I–V* measurements with the following sweeping voltage sequences: (1) 0 V → +6 V → 0 V (pristine RS from HRS to LRS, black line), (2) 0 V → +6 V → 0 V (maintaining LRS which was closely identical to the previous LRS curve, red line), (3) 0 V → −6 V → 0 V (reset switching from LRS to HRS, blue line), (4) 0 V → +6 V → 0 V (set switching from HRS to LRS again, green line). The difference between curve 2 and curve 4 in Fig. S3a demonstrates stability of both LRS and HRS, which were respectively obtained by set and reset switching. The stability of LRS was also supported by curves 4 and 5 in Fig. S3b, which were obtained by consecutive sweeps in the positive bias region (0 V → +6 V) after set switching. For each sample, there was a crossover of current levels at low bias region, which was often observed in oxide structures with non-volatile RS behaviours^18^,^33^,^34^,^35^. In spite of this crossover, our BFO nano-islands, similar to previously reported oxides^18^,^33^,^34^,^35^, showed non-volatile RS behaviours, where both LRS and HRS were repeatedly obtained whenever a reading voltage was applied after set and reset switching, respectively.

To confirm the nonvolatility of the RS observed in *I-V* characteristics, we performed pulsed voltage measurements on our BFO nano-islands. We observed reproducible switching and retention characteristics of both LRS and HRS obtained by applying pulses of +6 V and −6 V, respectively, for 100 ms. The retention behaviour measured at a reading voltage of +3 V showed that high resistance ratios of ~10^3^ were maintained after 10^3 ^s (see Supplementary Information Fig. S4a). Note that for the retention measurements, we successively deposited Pt top electrodes on the BFO nano-islands using e-beam evaporation before removing the AAO template, which provided good electrical contacts between the AFM tip and Pt/BFO/Nb:STO nano-structures (see Supplementary Information, Fig. S4b). These data demonstrate that our BFO nano-island device is a good candidate for selector-free non-volatile RS memory elements.

We should note that BFO nano-islands on SrRuO_3_ (SRO) electrodes showed switchable diode behaviours relevant to nearly symmetric ferroelectric hysteresis loops^20^. The BFO/SRO structures could not be used as ‘selector-free’ RS devices due to their low nonlinearity factor of ~4.6 at 0.6 V. In contrast, the BFO/Nb:STO structures, which have pinned downward polarization, show highly nonlinear bipolar RS behaviours. The strain gradient and flexoelectric effect caused by defective structures of BFO nano-islands on the Nb:STO substrates (Fig. 3) contribute to the formation of non-switchable polarization and the resulting high nonlinearity factor.

Figure 4b shows PFM phase, PFM amplitude, and C-AFM images obtained near a selected area (red-dashed circle) locally switched between HRS and LRS states. We observed very similar PFM phase of LRS to that of HRS, significantly smaller (dark contrast) PFM amplitude of LRS than those of HRS, and remarkably higher (dark contrast) local current of LRS to that of HRS. The HRS state is an initial state, while the LRS state is achieved by applying +4 V using the Pt/Ir coated AFM tip. The remarkable increase in local current of LRS compared to that of HRS cannot be induced by polarization reversal of the BFO nano-island because PFM phase images of both states are nearly identical. Instead, this may result from the movement of oxygen vacancies in the ferroelectric material, which can be controlled by external bias. It has been reported that oxygen vacancy migration^30^,^31^,^32^,^36^,^37^ in an oxide material plays an important role in its RS through modulation of the effective thickness of highly conductive oxide containing oxygen vacancies^21^. The concurrent decrease in PFM amplitude supports our RS model based on oxygen vacancy migration, which may diminish the polarization pinning effect.

The downward polarization of a BFO nano-island grown on Nb:STO can cause band bending (Fig. 4c), as well as formation of a Schottky barrier at the Pt/BFO interface where negative polarization charge at the tail repels conduction electrons to form a depletion layer^20^. Our Pt/BFO/Nb:STO device can be considered as a serial combination of a Schottky diode and a resistor^38^. In this case, the applied forward bias *V* (larger than k*T*/*q*) is expressed as follows.


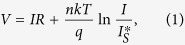


where *R* is the resistance of the bulk region in our BFO nano-island, *n* the ideality factor, k Boltzmann’s constant, *T* the temperature, *q* the electron charge, and 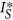 the saturation current of the Schottky diode at zero bias. Since the bulk resistance at HRS may be considerably high and most of the voltage drop appears at the bulk region in our BFO nano-island, we expect that *V* is linearly proportional to *I* in HRS, as shown in the inset of Fig. 4d. In contrast, low bulk resistance of our BFO nano-island at LRS gives rise to an exponential increase in *I* with *V*. By fitting with the standard semiconductor Schottky model, we can obtain a high ideality factor of 14 at LRS. Such a high ideality factor is often observed in nano-Schottky contacts which contain interfacial states such as structural defects and surface contamination^39^.

Based on these experimental observations, we can explain both positive-forward rectifying and bipolar RS behaviours of the individual BFO nano-island grown on Nb:STO as shown in Fig. 4e. Because BFO nano-islands were deposited at low oxygen pressure using a Bi-rich target material, an individual nano-island may contain a significant amount of oxygen vacancies. In the as-grown state, positively charged oxygen vacancies tend to accumulate near the top surface of BFO, as driven by a depolarization field of pinned downward polarization. The mosaic structure of BFO nano-islands promotes the migration of oxygen vacancies through grain boundaries where the ionic migration barrier is generally lower than within the grains. Therefore, the top region of a BFO nano-island can become a semiconductor with low resistance caused by the accumulated oxygen vacancies donating conduction electrons, while its bottom region remains a good insulator due to its lack of oxygen vacancies. The resultant high serial bulk resistance of the BFO nano-island, which may exceed the interfacial resistance of the Pt/BFO Schottky diode, leads to HRS of the Pt/BFO/Nb:STO capacitor structure. The linear dependence of the *I–V* curve at HRS in the inset of Fig. 4d supports our theory that the high bulk resistance of BFO governs the *I–V* behaviour at HRS, even though a downward Schottky diode is formed at the top Pt/BFO interface.

When a positive bias is applied to the top Pt/Ir-coated AFM tip, oxygen vacancies migrate downward and diffuse along the thickness direction of the BFO nano-island. As a result, most of the BFO bulk material (with excess oxygen vacancies) becomes a semiconductor whose resistance is much smaller than that of the downward Schottky diode at the top Pt/BFO interface. Therefore, a reduction in total resistance leads to switching from HRS to LRS, and the dominating Schottky diode results in positive-forward rectifying behaviour at LRS. By applying negative bias, oxygen vacancies can move back toward the top interface leading to recovery of HRS.

A typical memory element with bipolar RS has poor nonlinearity in its *I–V* curve, especially at its LRS; this can lead to sneak path problems through idle LRS elements in the cross-point array. When V_read_ is applied to a cell, 1/2 V_read_ is applied to neighbouring unselected cells. Because the poor nonlinearity of the *I–V* curve causes comparable current values of LRS at 1/2 V_read_ and V_read_ (which are much higher than that of HRS at V_read_), a significant amount of read current flows through a sneak path consisting of neighbouring unselected cells in the LRS, circumventing the selected cell in the HRS. This sneak path can be broadened as the number of unselected cells in the LRS is increased and the memory array circuit (N × M) is extended. It is important, therefore, to reduce the current level of unselected cells in LRS to solve this sneak path problem. Until now, suppression of the sneak path has been attempted by introducing a selection element stacked in a vertical structure on the memory element (1S1M)^7^,^9^,^11^,^12^,^13^,^15^,^16^,^17^,^18^,^19^,^40^: tunnelling device; *p*-*n* junction diode; threshold device; Schottky diode; complementary resistive switch. A good selection element should have large nonlinearity in the *I–V* curve of its LRS, providing a much smaller current value of LRS at 1/2 V_read_ than that at V_read_. As shown in Fig. 4a, our BFO nano-island device has a much smaller LRS current value at 1/2 V_read_ (green dot) than at V_read_ (blue dot), implying that it also acts as a good switch element which significantly reduces the sneak current.

**Figure 5:**
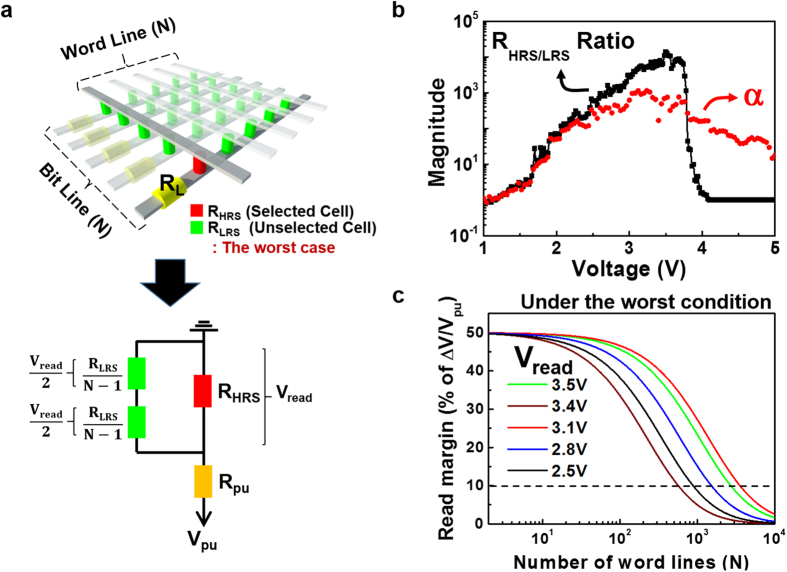
Possibly extended array of BFO memory cells. (**a**) Illustration of the cross-point memory array (N = M) and its simplified equivalent circuit in the read operation at the worst case. (**b**) R_HRS_/R_LRS_ ratio (black line) and nonlinearity factor (α, red line) as a function of V_read_, which are extracted from *I–V* curves at Fig. 4a. (**c**) Read margin ΔV/V_pu_ as a function of N at various V_read_ values.

The schematic of a typical N × N cross-point ReRAM array, where all unselected cells are at LRS (worst-case), is shown in Fig. 5a. A load resistor (R_L_) is connected at the end of each bit-line for reading stored data from memory cells at the cross-points. The resistance values of the memory cells are either R_LRS_ or R_HRS_, depending on the state of each cell. The LRS/HRS of the memory cell is identified by the electric current through R_L_ or the voltage drop across R_L_^41^. To read from the cross-point array, a worst-case read scheme, the so-called one bit-line pull-up scheme, is considered. Only one bit-line is pulled up, and all other bit lines are floating when each word line is selected^19^. This simplified equivalent circuit of a square N × N array with negligible line resistance is depicted in Fig. 4a^8^.

For a large array, the voltage across the parallel resistor network of the unselected word and bit lines (see R_LRS_/(N − 1) in Fig. 5a) is only half of the voltage across the selected cell (V_read_). To a first approximation, 2R_LRS_/(N − 1) at 1/2 V_read_ through the sneak path should not be less than R_LRS_ at V_read_ to ensure that a sufficient amount of read current flows through the selected cell. In other words, the LRS nonlinearity factor α is a primary factor determining the maximum array size, while the resistance ratio of HRS and LRS (R_HRS/LRS_) plays an important role in the optimization of the passive cross-point array. A more quantitative assessment on the read margin ∆V normalized to the pull-up voltage (V_pu_) can be calculated by solving the Kirchhoff equation as follows^19^.


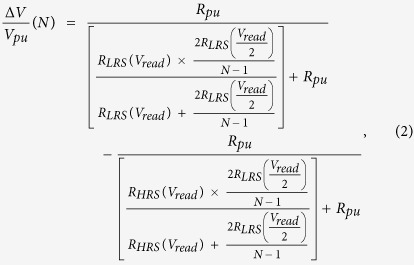


where R_pu_ is the resistance of R_L_ connected to the pulled-up bit line and set to R_LRS_ at V_read_ for maximum voltage swing (∆V)^8^.

The black line of Fig. 5b shows that R_HRS/LRS_ depends on V_read_ and reaches a maximum value of 14,000 at 3.5 V. From the red line of Fig. 5b, we find that the nonlinearity factor α of LRS, which is the ratio of R_LRS_ at 1/2 V_read_ to R_LRS_ at V_read_, has a maximum value of 1,100 at 3.1 V. We should note that the high nonlinearity factor of our selector-free memory cell is comparable to that reported previously in a 1S1M structure^19^.

Using the device parameters extracted from Figs 4a and 5b, the read margin (a percentage of ΔV/V_pu_) is obtained as a function of the number (N) of word lines (Fig. 5c). As we have assumed above, both R_HRS/LRS_ and α affect the read margin and thus the maximum cross-point array size with at least 10% read margin^7^. The maximum cross-point array size for our BFO nano-island is 2,830 at V_read_ = 3.5 V, where R_HRS/LRS_ shows the largest value. This can be further scaled up to 3,740 at V_read_ = 3.1 V, where the largest α value is observed. This implies that a V_read_ of 3.1 V leads us to a 10^7^-bit array based on our BFO nano-island monolithic 1S1M building block.

## Conclusions

In summary, we explored the local charge conduction and ferroelectric polarization in highly-oriented, mosaic-structured ferroelectric BFO nano-islands arrays grown on Nb:STO bottom electrodes by using conductive atomic force microscopy and piezoresponse force microscopy. Individual BFO nano-islands revealed pinned downward polarization, possibly caused by interfacial band bending and defect ions. We observed highly nonlinear rectifying and bistable current-voltage characteristics resulting from the coexistence of pinned polarization and oxygen vacancies in the Pt/BFO/Nb:STO structure, which were required for selector-free RS memory cells free from sneak path issues. A high resistance ratio between HRS and LRS of 4,420 and high nonlinearity factor of 1,100 were obtained at the read voltage of 3.1 V. The maximum array size could be scaled up to N = 3,740, which corresponds to a 10^7^ bit memory density. These results suggest that a BFO nano-island sandwiched between Pt electrode and Nb:STO substrate is a simple promising memory cell for replacing one selector–one memory structure.

## Methods

### Sample fabrication

AAO membranes with ~80 nm pore size were fabricated by a two-step anodization of electro-polished Al sheets^42^. The first anodization of Al sheet in 0.3 M H_2_C_2_O_4_ solution was conducted for 18 h at 5 °C. The anodized Al sheet was completely removed in an aqueous acid mixture of H_3_PO_4_ and CrO_3_ (6 wt% and 1.8 wt%, respectively) at 60 °C for 24 h. The second anodization was carried out for 3 min under the same conditions with the first anodization. Through this process, we fabricated the AAO membranes with a ~60 nm pore size. After the second anodization, a thin layer of polymethylmethacrylate (PMMA) C_4_ was coated on the surface of the AAO membrane and heated to 150 °C to evaporate the solvent. AAO membranes were detached from aluminum sheets in a saturated HgCl_2_ solution at room temperature. The PMMA C_4_ layer played an important role in handling and protecting the ultrathin AAO template. A barrier layer, which was a formed thin alumina layer at the bottom of pores during anodization, was removed with 5 wt% H_3_PO_4_ at 30 °C for 40 min. Because the PMMA C_4_ film protected the top of each pore, it was possible to selectively etch the bottom barrier layer. During the etching process, each pore was widened to 80 nm in diameter^43^. The AAO membrane was transferred onto a Nb:STO (100) single crystal substrate and then immersed in acetone for a few hours leading to complete removal of PMMA C_4_ from the AAO membrane. Finally, we obtained 180 nm-thick AAO membranes having excellent contact with the substrates.

Ferroelectric BFO nano-islands arrays were fabricated on Nb:STO substrates through AAO nano-templates using PLD (KrF excimer laser, λ = 248 nm) with an energy fluence of 320 mJ/cm^2^ and a repetition rate of 1 Hz. PLD was performed under an oxygen atmosphere of 20 mTorr at 450 °C using a Bi_1.1_Fe_1.0_O_3_ target. After BFO deposition, we pressed a piece of adhesive tape onto the template surface and lifted off the tape along with the AAO template, yielding an extended array of BFO nano-islands. Note that we also fabricated metal/ferroelectric/metal (MFM) structures for retention measurements. The MFM structures were obtained by successively depositing Pt top electrodes on the BFO nano-islands using e-beam evaporation before removing the AAO template.

### Characterization

Structural properties and morphologies of BFO nano-islands array were determined using high-resolution XRD (Bruker D8 discover) and AFM (Seiko SPA-300HV) topography at room temperature, respectively. High-resolution TEM images were recorded using a high-voltage electron microscope (JEM-ARM1300S, JEOL Ltd.) operating at 1,250 keV with a point resolution of 0.12 nm. Note that to avoid potential radiation-induced structural changes of the sample under the incident electron beam, samples were exposed for less than 1 min at an electron dose rate of 1.06 × 10^6^ electrons∙s^−1^∙nm^−2^ for high-resolution TEM image acquisition. The total electron dose on the sample was far less than the value required to trigger radiation-induced phase transition of aluminum hydroxide^44^.

GPA^45^ to measure the nanoscale strain field distribution was performed using GPA plug-in software (HREM Research Inc.) in the Gatan Microscopy Suite Software (Gatan Inc.). Morphologies of AAO were observed using field-effect scanning electron microscope (FE-SEM, TESCAN MIRA-II).

Local electrical properties of the BFO nano-islands were investigated using AFM in PFM mode with a lock-in amplifier (Stanford Research Systems SR830). For acquiring local piezoresponses and domain switching, AC voltage (V_rms_ = 1 V and frequency = 17 kHz) and DC voltage ranging from −5 V to +5 V were applied to a Pt/Ir-coated AFM tip (Nanosensors PPP-NCHPt, tip radius <30 nm) attached to a cantilever with a spring constant of 42 N/m and resonance frequency of 330 kHz. Local charge transport behaviour was measured using C-AFM with the same Pt/Ir-coated AFM tip, combined with a semiconductor analyser (HP4156B).

## Additional Information

**How to cite this article**: Jeon, J. H. *et al.* Selector-free resistive switching memory cell based on BiFeO_3_ nano-island showing high resistance ratio and nonlinearity factor. *Sci. Rep.* **6**, 23299; doi: 10.1038/srep23299 (2016).

## Supplementary Material

Supplementary Information
