# Supplementary material for: Selector-free resistive switching memory cell based on BiFeO3 nano-island showing high resistance ratio and nonlinearity factor
Source: Sci Rep. 2016 Mar 22;6:23299. doi: 10.1038/srep23299 (PMC4802337; doi:10.1038/srep23299)
Supplement: Supplementary Information [file srep23299-s1.doc]

**Supplementary Information**

**Selector-free resistive switching memory cell based on BiFeO_3_ nano-island showing high resistance ratio and nonlinearity factor**

**Ji Hoon Jeon^1^, Ho-Young Joo^2^, Young-Min Kim^3,4^, Duk Hyun Lee^1^, Jin-Soo Kim^1^, Yeon Soo Kim^1^, Taekjib Choi^2^,*, and Bae Ho Park^1^,***

^1^ Division of Quantum Phases & Devices, Department of Physics, Konkuk University, Seoul 143-701, Korea

^2^ HMC, Department of Nanotechnology and Advanced Materials Engineering, Sejong University, Seoul 143-747, Republic of Korea

^3^ IBS Center for Integrated Nanostructure Physics (CINAP), Institute for Basic Science, Sungkyunkwan University, Suwon 440-746, Republic of Korea

^4^ Department of Energy Science, Sungkyunkwan University, Suwon 440-746, Republic of Korea

^*^To whom correspondence should be addressed: baehpark@konkuk.ac.kr, tjchoi@sejong.ac.kr


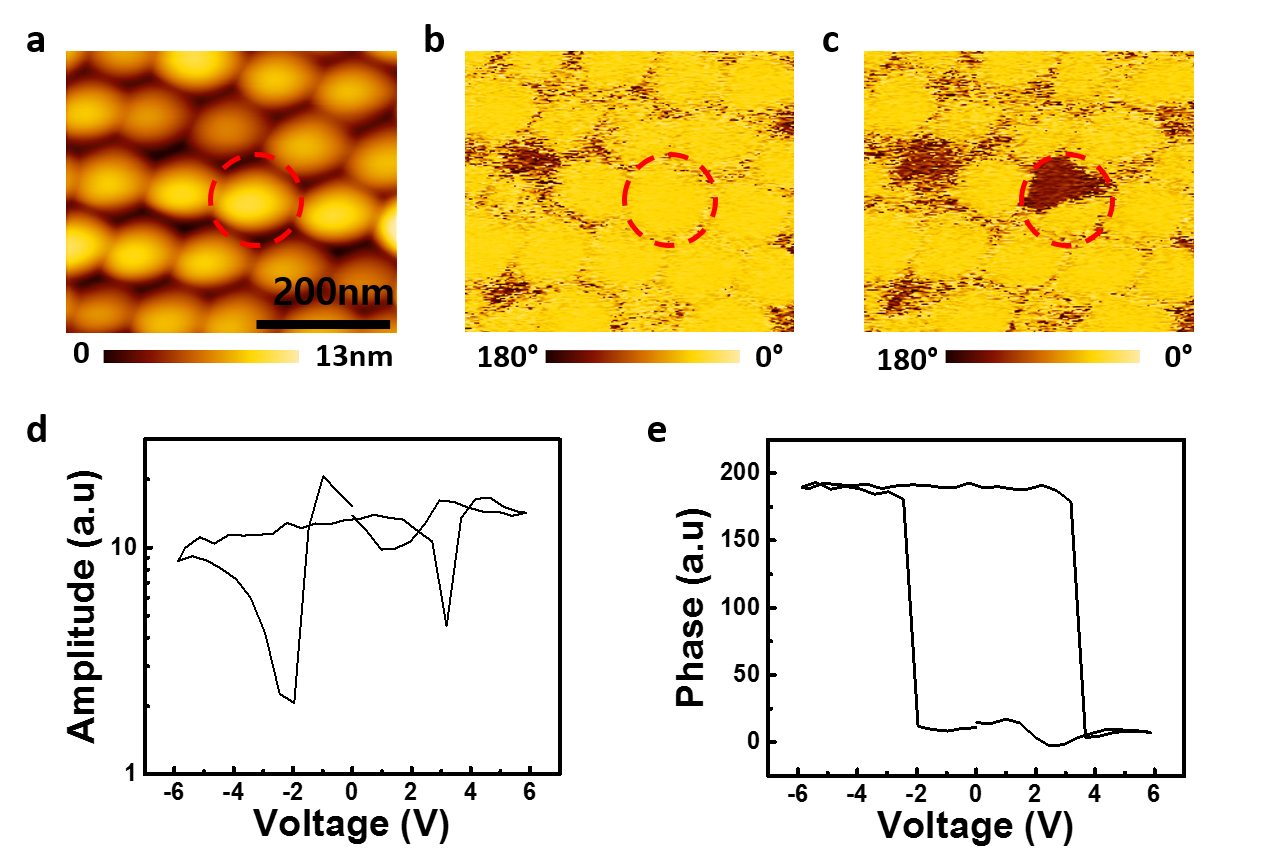


**Figure S1.** **PFM study and local ferroelectric properties of thicker BFO nano-islands (~50 nm thickness).** (a) AFM topography and (b) PFM phase images of as-grown BFO nano-islands with 50 nm thickness. (c) PFM phase image obtained after applying -6 V within the red dotted circle. Part of the individual nano-island inside the red dotted circle is switched, indicating that the BFO nano-island may have a multi-domain structure. Dependence of (d) PFM amplitude and (e) PFM phase on applied voltage sweeping between -6 V and +6 V, which were obtained at a single BFO nano-island within the red dotted circle. The butterfly shape of the amplitude loop and 180° change in the phase loop can be ascribed to the polarization reversal in the thicker BFO nano-island.

**c**


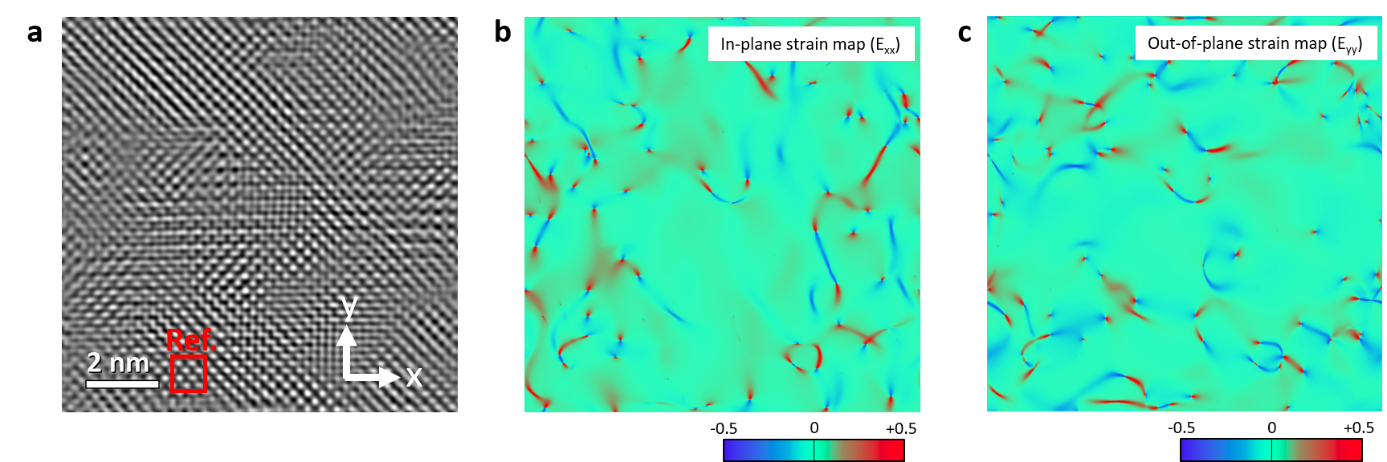


**Figure S2.** **Geometric phase analysis to obtain strain field maps and distributions of defect cores.** (a) High-resolution TEM image shown in Figure 3b. The x and y directions are chosen to be the in-plane and out-of-plane directions of the [100]-oriented pseudo-cubic BFO, respectively. Strain field maps of (b) E_xx_ and (c) E_yy_ indicating strain behaviours relative to the reference area along the in-plane (x) and the out-of-plane (y) directions, respectively. The reference area is marked by a red box in (a) and the colour bars in (b) and (c) indicate the full strain range from -0.5 and +0.5.


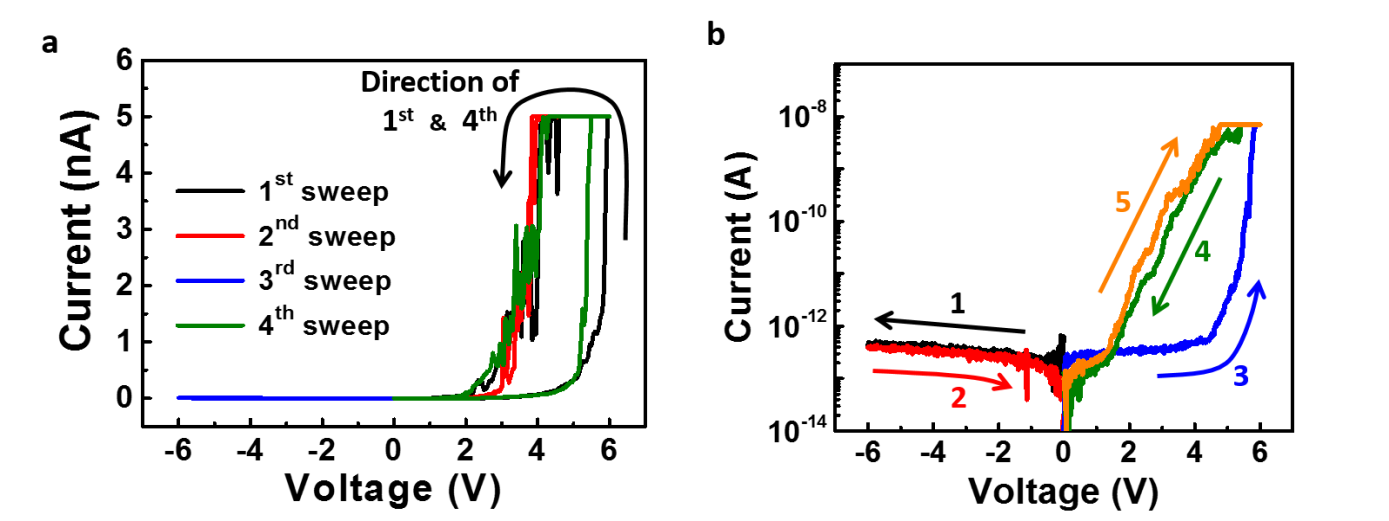


**Figure S3.** **The cyclic *I-V* characteristics of BFO nano-islands on Nb:STO substrate.** (a) Local *I-V* curves (linear scale) of a BFO nano-island grown on Nb:STO, which are obtained by applying a sequence of cyclic sweeps: (1) 0 V 🡪 +6 V 🡪 0 V (black line), (2) 0 V 🡪 +6 V 🡪 0 V (red line), (3) 0 V 🡪 -6 V 🡪 0 V (blue line), (4) 0 V 🡪 +6 V 🡪 0 V (green line). (b) Local *I-V* curves (log scale) of another BFO nano-island, which are obtained by applying a sequence of sweeping voltages: (1) 0 V 🡪 -6 V (black line), (2) -6 V 🡪 0 V (red line), (3) 0 V 🡪 +6 V (blue line), (4) +6 V 🡪 0 V (green line), (5) 0 V 🡪 +6 V (yellow line). Measurements were carried out using a Pt/Ir-coated AFM tip as a top electrode. Note that measured BFO nano-islands were randomly selected on the nano-island array.


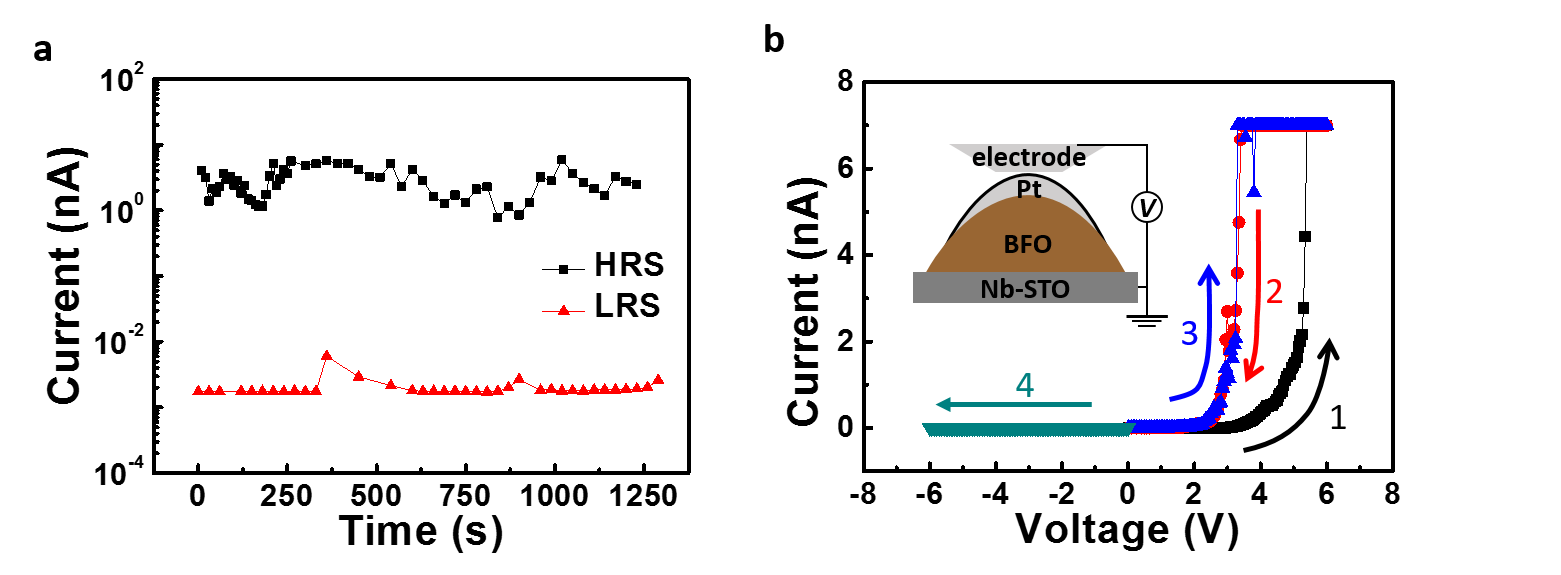


**Figure S4.** **The retention and *I-V* characteristics of Pt / BFO nano-island / Nb:STO capacitor.** (a) The retention characteristics of Pt-capped BFO nano-islands grown on Nb:STO. Black and red lines display the change of current values in HRS and LRS, respectively. The retention behaviour was measured at a reading voltage of +3 V, and the high resistance ratio is maintained for 10^3^ s. (b) *I–V* characteristics of an individual Pt/BFO/Nb:STO nano-capacitor. The numbers and arrows indicate the sequence of sweeping voltages: (1) 0 V 🡪 +6 V (black line), (2) +6 V 🡪 +0 V (red line), (3) 0 V 🡪 +6 V (blue line), (4) 0 V 🡪 -6 V (green line). The inset shows the schematic illustration of the measurement geometry.
